# Interoceptive awareness: MBSR training alters information processing of salience network

**DOI:** 10.3389/fnbeh.2023.1008086

**Published:** 2023-03-21

**Authors:** Shiao-Fei Guu, Yi-Ping Chao, Feng-Ying Huang, Yu-Ting Cheng, Hei-Yin Hydra Ng, Chia-Fen Hsu, Chun-Hsiang Chuang, Chih-Mao Huang, Changwei W. Wu

**Affiliations:** ^1^Graduate Institute of Mind, Brain and Consciousness, Taipei Medical University, Taipei, Taiwan; ^2^Department of Computer Science and Information Engineering, Chang Gung University, Taoyuan, Taiwan; ^3^Department of Otorhinolaryngology-Head and Neck Surgery, Chang Gung Memorial Hospital, Taoyuan, Taiwan; ^4^Department of Education, National Taipei University of Education, Taipei, Taiwan; ^5^Clinical Mental Health Counseling Program, Johns Hopkins University, Baltimore, MD, United States; ^6^Department of Educational Psychology and Counseling, College of Education, National Tsing Hua University, Hsinchu, Taiwan; ^7^Research Center for Education and Mind Sciences, College of Education, National Tsing Hua University, Hsinchu, Taiwan; ^8^Graduate Institute of Behavioral Sciences, Chang Gung University, Taoyuan, Taiwan; ^9^Department of Child Psychiatry, Chang Gung Memorial Hospital at Linkou, Taoyuan, Taiwan; ^10^Department of Biological Science and Technology, National Yang Ming Chiao Tung University, Hsinchu, Taiwan; ^11^Center for Intelligent Drug Systems and Smart Bio-Devices (IDS2B), National Yang Ming Chiao Tung University, Hsinchu, Taiwan; ^12^Brain and Consciousness Research Center, Shuang Ho Hospital-Taipei Medical University, New Taipei, Taiwan

**Keywords:** mindfulness, mindfulness-based stress reduction (MBSR), functional magnetic resonance imaging (fMRI), functional connectivity, regional homogeneity, interoceptive awareness, salience network

## Abstract

Mindfulness refers to a mental state of awareness of internal experience without judgment. Studies have suggested that each mindfulness practice may involve a unique mental state, but the underlying neurophysiological mechanisms remain unknown. Here we examined how distinct mindfulness practices after mindfulness-based intervention alter brain functionality. Specifically, we investigated the functional alterations of the salience network (SN) using functional magnetic resonance imaging (fMRI) among the two interoceptive mindfulness practices—breathing and body scan—associated with interoceptive awareness in fixed attention and shifted attention, respectively. Long-distance functional connectivity (FC) and regional homogeneity (ReHo) approaches were applied to measure distant and local neural information processing across various mental states. We hypothesized that mindful breathing and body scan would yield a unique information processing pattern in terms of long-range and local functional connectivity (FC). A total of 18 meditation-naïve participants were enrolled in an 8-week mindfulness-based stress reduction (MBSR) program alongside a waitlist control group (*n* = 14), with both groups undergoing multiple fMRI sessions during breathing, body scan and resting state for comparison. We demonstrated that two mindfulness practices affect both the long-distance FC_*SN*_ and the local ReHo, only apparent after the MBSR program. Three functional distinctions between the mindfulness practices and the resting state are noted: (1) distant SN connectivity to occipital regions increased during the breathing practice (fixed attention), whereas the SN increased connection with the frontal/central gyri during the body scan (shifting attention); (2) local ReHo increased only in the parietal lobe during the body scan (shifting attention); (3) distant and local connections turned into a positive correlation only during the mindfulness practices after the MBSR training, indicating a global enhancement of the SN information processing during mindfulness practices. Though with limited sample size, the functional specificity of mindfulness practices offers a potential research direction on neuroimaging of mindfulness, awaiting further studies for verification.

## 1. Introduction

Mindfulness refers to a mental state of awareness of internal and external experiences without judgment (Kabat-Zinn, 1994). Mindfulness-based stress reduction (MBSR) is the most widely studied form of mindfulness-based intervention. MBSR was designed to enhance a mindfulness practitioner’s abilities to retain awareness in the present, interpret intra- and interpersonal situations with clarity, and respond appropriately to adversity (Santorelli et al., 2017). Numerous studies have reported the benefits of MBSR, such as reduced anxiety and depression, enhanced emotional regulation, alleviation of sleep disturbances, improved quality of life, and even postponed dementia onset among mindfulness practitioners (Davidson et al., 2003; Goldin and Gross, 2010; Larouche et al., 2015; Huang et al., 2019).

Using functional magnetic resonance imaging (fMRI), recent meta-analysis disclosed that mindfulness trainings alter the brain connectivity of multiple brain networks, associated with the cognitive performances of attention control, pain relief and emotion regulation (Sezer et al., 2022). These beneficial outcomes and brain-network changes after the mindfulness training may originate from the “being mode” mindset (i.e., non-attachment, non-striving, and an accepting attitude) and the persistent practice of core techniques during the 8-week MBSR program, including breathing, body scan, yoga, loving-kindness, sitting meditation, and open monitoring (Kabat-Zinn, 1990, 2003). Although these elementary mindfulness practices are not usually differentiated from the entirety of MBSR package, studies have demonstrated that mindfulness practices may involve a certain level of cognition and underlying peripheral nervous system. For example, Lumma et al. demonstrated that both the increase in heart rate and the reduction in high-frequency heart rate variability (HRV) reported after meditation training are more prominent after loving-kindness than after breathing practice (Lumma et al., 2015). Kok et al. compared the state changes recorded through self-assessed psychological measures after presence (breathing and body scan), loving-kindness, and observing-thoughts meditation, concluding that each technique is characterized by its own distinct short-term psychological fingerprint (Kok and Singer, 2017). From the viewpoint of brain functions, Brewer et al. demonstrated the elevated connectivity between dorsal anterior cingulate cortex (dACC) and posterior cingulate cortex (PCC) only occurred in the choiceless awareness, rather than concentration or loving-kindness meditations, among long-term meditators (Brewer et al., 2011), implying the distinction of goal-directed attention levels among different mindfulness practices. Moreover, Fujino et al. reported reduced ventral-striatum connectivity during the open monitoring meditation but increased connectivity in the same regions during the focused attention meditation (Fujino et al., 2018), inferring the reduced habitual behavior and enhanced memory function during the open-monitoring meditation. At the current stage, the neurophysiological specificity of distinctive mindfulness practices remains largely unknown. Therefore, identifying the discrepancies in the brain-network organizations yielded by distinct mindfulness practices can provide further insights into how mindfulness-based intervention shapes brain functionality and alters behaviors.

To achieve this goal, we determined to investigate the two fundamental mindfulness practices—the mindful breathing and the body scan. These two practices typically require the use of interoceptive awareness with increased vagal tone (Gerritsen and Band, 2018). By placing attention on bodily sensations, these practices help to anchor the mind in the present moment, thereby facilitating relaxation without entanglement in affective ruminations (Gibson, 2019). Although these two practices are classified within the same broad category, each has its own unique features. Breathing meditation entails fixed attention on the bodily sensations associated with breathing (e.g., the passage of airflow through the nostrils, contraction of the abdomen), whereas during the body scan practice, participants are instructed to shift their attention toward certain body parts sequentially (Malinowski, 2013; Gibson, 2019). In subjective reports, some meditation participants prefer the body scan practice to breathing because it is less monotonous and thus relaxing, whereas others express a preference for breathing precisely because of its emphasis on a single target for interoceptive awareness without the need to constantly shift attention to other objects. The distinct features and subjective evaluations of adaptive interoceptive attentional styles observed between breathing mediation and body scan lead to the speculation that these two mindfulness practices would involve in distributed but dissociable neurocognitive mechanisms (Tomasino and Fabbro, 2016; Gibson, 2019). In our previous work with objective measures, we conducted electroencephalography (EEG) recordings in both practices (breathing and body scan) before and after the MBSR program, and the results indicated reduced EEG Delta power during the breathing but not the body scan (Ng et al., 2021), suggesting a higher vigilance level in the breathing practice among novice practitioners. Accordingly, we speculate that even in individuals with similar levels of attention and introspection, different mindfulness strategies (breathing and body scan) can lead to distinctive brain-connectivity patterns.

The resting-state functional connectivity (FC) is the neuroimaging technique to extract the spatiotemporal features of spontaneous synchronizations without external stimuli (Biswal et al., 1995), and the long-distance FC elucidate neural information processing across various mental states (Haase et al., 2015; Jao et al., 2016). To understand the neural circuitry underlying mindfulness, the FC approach has been widely applied to topics ranging from the mindfulness trait (Parkinson et al., 2019) and mindfulness intervention (Doll et al., 2015; Huang et al., 2020) to clinical pathologies (Zhao et al., 2019; Fam et al., 2020). For example, Kral et al. demonstrated that FC between the PCC and the dorsolateral prefrontal cortex (DLPFC) continually increased, even after the 8 weeks of MBSR program (Kral et al., 2019), which implies a plausible neuroimaging indication of altered thought processes in a wandering mind. However, most of the FC studies examined temporal correlations among brain regions distant away from each other, but the local information processing in brain (i.e., neural communications within a 2–10 mm radius), in which interneurons are clustered and exhibit a common resonance mode (Gray, 1999), has often been overlooked. To this viewpoint, we further hypothesize that the two mindfulness practices, breathing and body scan, may display different information processing patterns in terms of long-range and local connectivity. To account for the FC in both distant and local spatial scales, this study adopted an additional functional index of regional homogeneity (ReHo). ReHo is a measurement of intraregional communication within a set of nearest-neighbor voxels, reflecting local synchronizations or local connectivity of brain spontaneous activities (Zang et al., 2004). Previous studies found the positive correlation between ReHo and glucose metabolism (Nugent et al., 2014; Aiello et al., 2015), and investigators commonly used ReHo to evaluate brain functions in clinical cases, such as depression or migraine (Iwabuchi et al., 2015; Cui et al., 2022; Lin et al., 2022). Recently, we analyzed the local and distant connectivity (ReHo and FC, respectively) simultaneously across different sleep stages, and the observations of increased ReHo and declined FC during the sleep stage 2 indicated the altered brain functionality toward local processing, rather than long-distance communications in wakefulness (Kung et al., 2021). Therefore, we hypothesized that the information processing pattern, regarding both local and distant connectivity at the same time, changes during mindfulness practices and/or after training. To date, only four studies have utilized the ReHo index to evaluate the brain functional changes in mindfulness programs (Kong et al., 2015; Yang et al., 2016; Xiao et al., 2019; Zhao et al., 2019). Two out of the four studies based on mindfulness interventions indicated the declined ReHo in the dorsal anterior cingulate cortex (dACC) and the increased ReHo in the right superior parietal lobule after the 8-week MBSR program (Xiao et al., 2019; Zhao et al., 2019), demonstrating the possibility of changed local connectivity after the mindfulness training. Additionally, Yang et al. reported non-significant ReHo changes after a 40-day mindfulness program (Yang et al., 2016). Because of the inconsistency in the results of these ReHo studies, it is unclear whether mindfulness practices modulate the local connectivity, along with the distant connectivity at the same time (the underlying information processing). To test the brain-network reorganization in mindfulness, we postulated three possible scenarios: (1) both FC and ReHo increase, indicating an overall enhancement of information processing irrelevant of spatial scales; (2) FC increases, but ReHo remains the same, implying an enhancement of long-distance communication alone (between-network connectivity); and (3) FC increase, but ReHo decreases, signifying a shift in resource allocation from local to distant information processing. By examining FC at both the local and distant levels, the differences in brain information processing can be identified between the normal resting state and the mindfulness practices.

We examined the distinct functional characteristics of different mindfulness practices, and the longitudinal training effect (before and after the MBSR program). Hence, a pre- and post-MBSR-intervention design was implemented to investigate the brain functionality post mindfulness training, and to account for the possibility that meditation-naïve participants may be unable to differentiate between breathing and body scan practices. Regarding the network specificity of FC, studies have indicated that interoceptive awareness (breathing and body scan) is associated with the salience network (SN), especially on dACC and insula (Singer et al., 2004; Sevinc et al., 2018; Gibson, 2019).

## 2. Materials and methods

### 2.1. Participants

A total of 33 healthy Taiwanese adults, aged 28–68 years, participated in this study. Candidates with prior meditation experiences, or with a history of cardiovascular diseases, terminal illness, or surgery involving metallic implants, were excluded from the cohort. The cohort was randomly divided into an MBSR group (MBSR; *n* = 18) and a waitlist control group (CTRL). One CTRL group participant was unable to attend the second data collection session and was thus excluded from the final analysis (*n* = 14). Prior to the experiment, written informed consents was obtained from each participant, and the study protocol was approved by the Joint Internal Review Board of Taipei Medical University (N201905049).

### 2.2. Intervention: MBSR and CTRL

Each participant completed questionnaires and MRI experiments twice. The first data collection session was regarded as the baseline (PRE), and the second data collection session was held 8 weeks later (POST). The MBSR group was enrolled in an 8-week MBSR training program (Kabat-Zinn, 1994; Santorelli et al., 2017). The MBSR course was taught by a licensed MBSR instructor. The classes schedule consisted of eight 2.5-h weekly classes in addition to a single day-long mindfulness workshop. These weekly classes involved the development of several mindfulness-related skills, dialogue, reflection on the home-based mindfulness practice, and formal practice sessions. The participants were assigned daily home-based mindfulness practices, which comprised both formal and informal meditation activities. The formal activities included body scan, mindful breathing, sitting meditation, mindful yoga, walking meditation, mountain or lake meditation, and loving kindness, required 45 min to complete each day. The participants were required to complete a practice and information sheet. The CTRL group was instructed to continue their usual life activities but not engaged in any mindfulness-related activities (including religious activities) during the 8 weeks. After the POST experiment, the CTRL group received an additional MBSR program as compensation.

### 2.3. Experimental procedure

The participants completed three questionnaires: (1) the Five Facet Mindfulness Questionnaire (FFMQ) (Baer et al., 2006); (2) the Difficulties in Emotion Regulation Scale (DERS) (Gratz and Roemer, 2004); (3) the Pittsburg Sleep Quality Index (PSQI) (Buysse et al., 1989). An fMRI protocol was performed using a 3-T PRISMA scanner (Siemens, Erlangen, Germany) at National Taiwan University. Structural images were obtained using a high-resolution magnetization prepared rapid acquisition gradient echo (MPRAGE) sequence (repetition time [TR] = 2,000 ms; echo time [TE] = 2.28 ms; flip angle [FA] = 8°; image dimensions: 192 × 256 × 256; voxel resolution: 1 × 1 × 1 mm^3^). Functional images were obtained using a gradient-echo echo-planer imaging (GE-EPI) sequence (TR: 2,000 ms; TE: 32 ms; FA: 77°; image dimensions: 64 × 64 × 33; voxel dimensions: 3.4 × 3.4 × 3.4 mm^3^). The scanning session consisted of one T_1_-weighted anatomical scan, followed by three functional scans. During the resting-state (*Resting*) scan (5 min, 150 measurements), the participants were instructed to lie still with their eyes closed, not to fall into sleep, not dwell on one singular train of thoughts, and not to perform any mindfulness practices. Then, another 5-min (150 measurements) mindful breathing (*Breath*) was conducted, during which the participants were asked to lay still with their eyes closed, and to focus on the sensation of the breath passing through the nostrils and the philtrum areas. Finally, a 5-min (150 measurements) body scan practice (*BodyScan*), was performed, during which the participants were instructed to lie still with eyes closed but to this time focus on bodily sensations they were felt and remained aware of these sensations without analysis or judgements to them. The data of this study will be available on request from the corresponding author.

### 2.4. fMRI analysis

Functional imaging data pre-processing was performed using IClinfMRI (Hsu et al., 2018) in MATLAB R2014b (Mathworks Inc., Natick, MA) and analysis of functional neuroimaging (AFNI) (Cox, 1996). The procedure involved slice-timing correction, alignment with the anatomical T_1_-weighted image, head motion correction (censoring criteria: maximum displacement of > 3 mm or delta displacement > 0.5 mm, and the maximum volume number above the criteria was 21), de-spiking, linear detrending, nuisance regression (the used regressors included motion parameters, white matter and cerebrospinal fluid), bandpass filtering with a range of 0.01–0.08 Hz, spatial smoothing (Gaussian kernel: FWHM = 4 mm), and spatial normalization to the standard Montreal Neurological Institute (MNI)-152 template (voxel size: 2 mm^3^ × 2 mm^3^ × 2 mm^3^). The fMRI data of two participants (both in MBSR:PRE) in the *Breath* session were neglected from further analysis, one because of equipment malfunctioning on the day of scanning, and the other because of excessive head motion. Voxel-wise seed-based correlation analysis (seeding with 4-mm radius) was employed to quantify the FC strengths of the SN. In the SN, the seed locations were prescribed from the bilateral dorsal anterior cingulate cortex (dACC, MNI: ± 6, 18, 28) (van der Werff et al., 2013), and the bilateral anterior insula (MNI: ± 38, 26, –5) (Goveas et al., 2013). The voxel-wise correlation between the seeds and the whole brain were calculated using Fisher’s Z transformation to standardize them for further statistical analysis. Regarding the local connectivity of ReHo, Kendall’s coefficient of concordance (KCC), a measurement of the correlation between each voxel and its 93 neighboring voxels (radius = 2.9 mm) over the time course, was calculated with 3dReHo in AFNI (Taylor and Saad, 2013). Finally, an MNI template was applied as the regional mask to generate a standardized image of the entire brain for statistical analysis.

### 2.5. Statistical analysis

Statistical analysis was performed using GraphPad Prism (version 5.00 for Windows, GraphPad Software, San Diego, CA, United States), and the significance level was set as *p* < 0.05 with multiple comparison of the false-discovery-rate (FDR) method. Questionnaire scores (FFMQ, DERS and PSQI) were analyzed with a 2-way mixed Analysis of Variance (group × time), followed by *post hoc* tests with FDR correction. For the fMRI indices, POST–PRE paired comparisons of FC and ReHo were performed based on Threshold Free Cluster Enhancement (TFCE: 5000 permutations) (Smith and Nichols, 2009) for both MBSR and CTRL groups using the FSL randomise (Winkler et al., 2014). From TFCE results, we prescribed multiple regions of interest (ROIs) from the nearest Yeo brain template (400 parcellations, noted as Yeo_400_) to extract FC and ReHo values for ROI analysis (Yeo et al., 2011; Schaefer et al., 2017). Subsequently, we performed the same 2-way mixed ANOVA (group × time) and *post hoc* tests with FDR correction. To unveil the time/training effect in the MBSR group, an additional one-way repeated-measure ANOVA (rm-ANOVA) with FDR correction was also applied to the POST-PRE differences of connectivity indices (ΔFC and ΔReHo) across the 3 conditions. At last, Pearson correlation analysis was used for two evaluations: (1) [time effect] to calculate the correlation between the POST–PRE changes of questionnaire scores (ΔFFMQ and its five sub-scales, ΔDERS, and ΔPSQI) and the those of functional indices (ΔFC and ΔReHo); and (2): [practice effect] to estimate whether the correlations between FC and ReHo (the hypothetical information processing) would change across the three conditions (*Resting, Breath* and *BodyScan*), before and after the training in the MBSR group.

## 3. Results

No significant differences in age, sex, or educational level were noted between the MBSR and CTRL groups (Table 1). Regarding a potential MBSR training effect, Table 2 presents that significant group × time interactions were found in FFMQ (*F_1,30_* = 13.28, *p* = 0.001) and DERS (*F_1,30_* = 4.12, *p* = 0.05), but PSQI only exhibited significant time effect (*F_1,30_* = 4.88, *p* = 0.035) without interaction. The *post hoc* tests exhibited that significance POST-PRE differences in the MBSR group, but not in the CTRL group. In the MBSR group, all participants exhibited significantly higher FFMQ scores (*t*_17_ = 5.32, *p* < 0.001), in each of the five dimensions (i.e., observing, describing, acting with awareness, non-judgment, and non-reaction) in the post-MBSR assessments (compared with PRE). The MBSR group members exhibited significant decreases in DERS (*t*_17_ = 2.48, *p* = 0.047), indicating improvements in emotional regulation. By contrast, in the CTRL group, no such changes in questionnaire scores between the PRE and POST data collection sessions were noted.

**TABLE 1:** Demographics.

	MBSR	CTRL	Statistical analysis
Sample size	18	14	
Sex (F/M)	17/1	11/3	*p* = 0.465 (χ_1_^2^ = 0.533, n.s.)
Age (years)	47.50 (9.6)	45.87 (8.1)	*p* = 0.604 (t_31_ = 0.524, n.s.)
Education (years)	16.89 (2.0)	17.60 (2.5)	*p* = 0.377 (t_31_ = 0.897, n.s.)

Mean (standard deviation). *p < 0.05. n.s.: non-significant.

**TABLE 2:** Intervention effects of questionnaire scores between MBSR and CTRL groups.

Questionnaire	MBSR		CTRL		Group × Time interaction	
	**Pre-test**	**Post-test**	**Pre-test**	**Post-test**	***F***	***p***
FFMQ	114.90 (16.9)	142.60 (23.2)	116.60 (17.1)	115.90 (15.2)	13.3	0.001**
(a) Observe	23.28 (4.7)	30.22 (4.9)	23.71 (4.9)	24.93 (3.9)	11.3	0.002**
(b) Describe	22.94 (5.5)	26.22 (7.1)	24.21 (4.7)	24.07 (4.6)	5.6	0.025*
(c) Act with Awareness	25.11 (4.7)	29.89 (6.4)	26.07 (5.3)	24.50 (6.0)	8.7	0.006**
(d) Non-judge	23.94 (5.3)	32.33 (5.8)	22.64 (4.7)	22.79 (6.1)	15.2	<0.001***
(e) Non-react	19.67 (3.6)	23.89 (5.3)	19.93 (4.4)	19.64 (3.1)	8.8	0.006**
DERS	97.78 (20.7)	84.72 (23.2)	95.50 (23.2)	99.93 (16.1)	4.1	0.05*
PSQI	8.39 (3.9)	6.11 (3.8)	5.57 (2.9)	5.00 (2.8)	1.8	0.196^n.s.^

Mean (standard deviation). *p < 0.05. **p < 0.01. ***p < 0.001. n.s.: non-significant.

**FIGURE 1:**
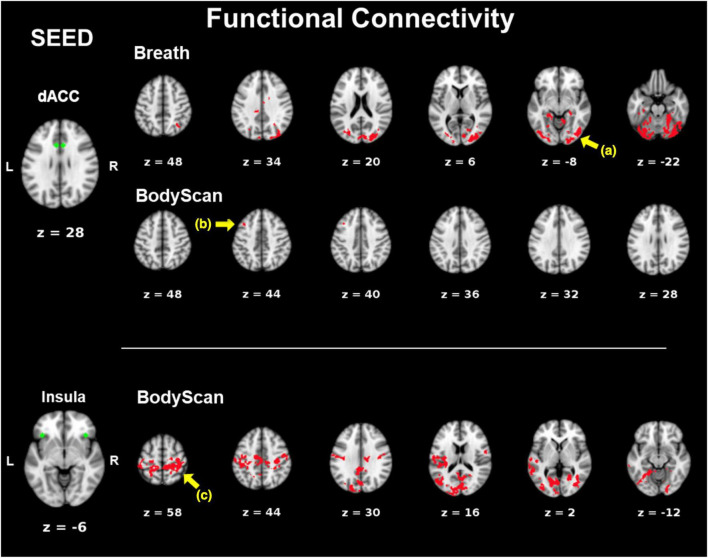
POST–PRE comparison of FC in the MBSR group using TFCE. Seeds are marked in green. Areas of significant post-pre differences (*p* < 0.05, FDR-corrected) in FC are marked in red. **(a)** The bilateral occipital lobe; **(b)** left middle frontal gyrus; **(c)** the precentral and post central gyri.

**FIGURE 2:**
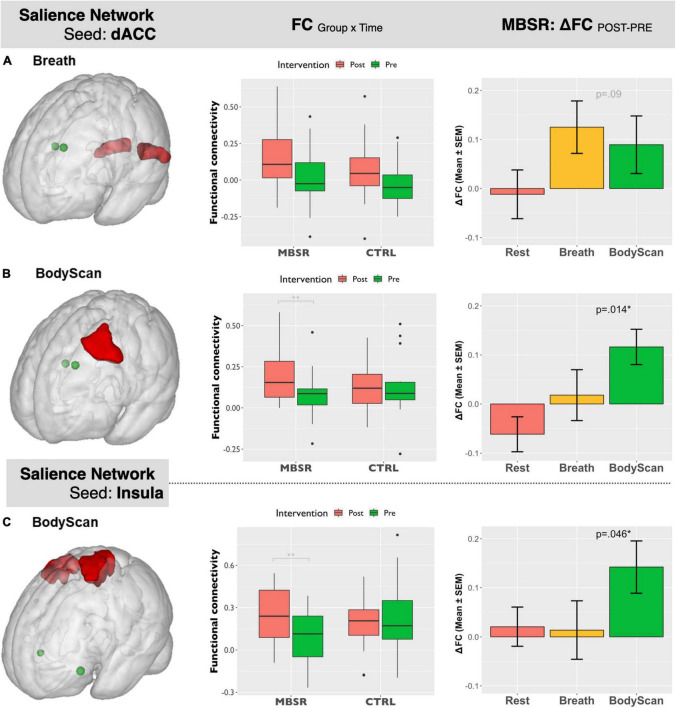
Salient Network-seeding at dACC: **(A)** (left) ROI of bilateral occipital lobe extracted from Yeo_400_ template, (middle): the ROI-based boxplot presenting the group × time interactions, and (right) the repeated-measure ANOVA of the FC changes across the 3 conditions (Rest, Breath, and BodyScan) in the MBSR group; **(B)** (left) ROI of the left middle frontal gyrus from Yeo_400_ template, (middle): the ROI-based boxplot presenting the group × time interactions, and (right) the repeated-measure ANOVA of the FC changes in the MBSR group. Salient Network-seeding at Insula: **(C)** (left) ROI of bilateral pre/postcentral gyri from Yeo_400_ template, (middle): the ROI-based boxplot presenting the group × time interactions, and (right) the repeated-measure ANOVA of the FC changes in the MBSR group. The error bars in the third column stands for standard error of the means (SEM).

**FIGURE 3:**
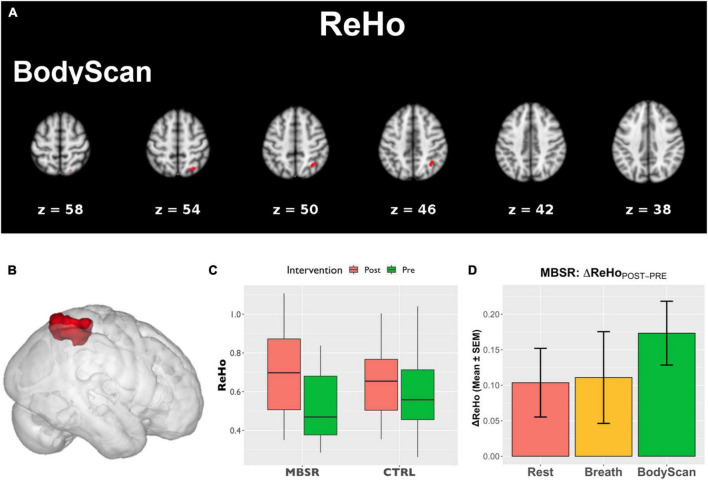
The analysis of ReHo. **(A)** Areas that showed POST–PRE differences of ReHo in TFCE (*p* < 0.05, FDR-corrected); **(B)** ROI of the right angular gyrus and parietal lobe extracted from Yeo_400_ template; **(C)** the ROI-based boxplot presenting the group × time interactions; **(D)** the repeated-measure ANOVA of the ReHo changes in the MBSR group, and the error bars in **(D)** stands for standard error of the means (SEM).

Figure 1 shows the SN-related FC maps exhibiting significant POST–PRE differences across the *Breath* and *BodyScan* conditions (TFCE, *FDR-corrected p* < 0.05), and the significant FC changes were only observed in the MBSR group, not in the CTRL group. During the *Breath* practice, the seed dACC yielded increased FC in the bilateral occipital regions, including the bilateral fusiform, lingual gyri, and the ROI findings were presented in Figure 2A (Yeo_400_ 2/3/4/202/203/204, group × time interaction: *F_1,30_* = 0.08, *p* = 0.776; rm-ANOVA across POST-PRE changes of ΔFC_Rest_, ΔFC_Breath_, ΔFC_BodyScan_: *F_2,30_* = 2.53, *p* = 0.096). During the *BodyScan* practice, the left middle frontal gyrus (lMFG) exhibited increased FC with the bilateral dACC (Figure 2B: Yeo_400_ 181, group × time interaction: *F_1,30_* = 4.30, *p* = 0.012; rm-ANOVA: *F_2,30_* = 4.91, *p* = 0.014). With seeding at bilateral anterior insula, the SN exhibited increased FC with the sensorimotor network (SMN) during *BodyScan* after training (Figure 2C: Yeo_400_ 33/34/35/230/231/232/233, group × time interaction: *F_1,30_* = 3.21, *p* = 0.08; rm-ANOVA: *F_2,30_* = 3.41, *p* = 0.046). An increased FC of the SN-related regions was noted during the mindfulness practices (the third column of Figure 2), and such FC increase was not apparent when participants were in the resting state. Regards with ReHo, the right angular gyrus and superior parietal lobe (Yeo_400_ 268/269/270) exhibited POST-PRE difference in the MBSR group during the *BodyScan* (Figures 3A, B), but it did not show significant group × time interaction (Figure 3C, *F*_1,30_ = 2.14, *p* = 0.15, with rm-ANOVA: *F_2,30_* = 0.33, *p* = 0.72, Figure 3D). In general, the results for both FC and ReHo suggest increased long-distance connectivity and mostly unchanged local connectivity after the MBSR training.

**FIGURE 4:**
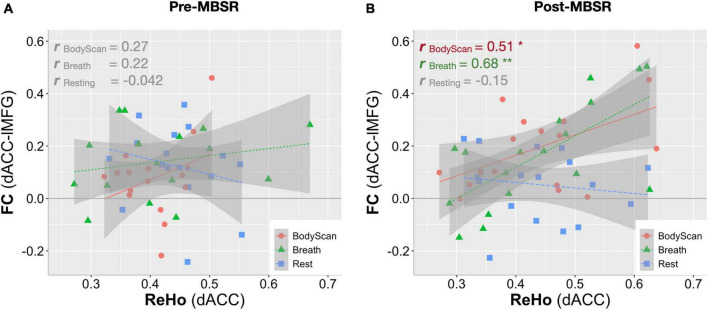
Correlation analysis between FC and ReHo for the left middle frontal gyrus in each of the 3 conditions (Rest, Breath and BodyScan) of the MBSR group. **(A)** Before the MBSR training, no significant FC-ReHo relationship was observed. **(B)** After the MBSR training, post-intervention FC_dACC_ exhibited a positive relationship with ReHo during both the breathing and the body scan, indicating a changed pattern of information processing in the salience network.

To demonstrate the time effect, we estimate the association between questionnaire score changes and connectivity changes (ΔFC and ΔReHo). In the *Breath* condition, ΔFC_*dACC–occipital*_ (Figure 2A) and ΔReHo_*angular*_ (Figure 3B) both exhibited positive correlation with ΔObserve (*r* = 0.49, *p* = 0.005; and *r* = 0.50, *p* = 0.005, respectively). In the *BodyScan* condition, ΔFC_*Insula–SMN*_ (Figure 2C) exhibited positive correlation with ΔObserve (*r* = 0.40, *p* = 0.025), and ΔReHo_*lMFG*_ exhibited negative correlation with ΔDERS (*r* = –0.39, *p* = 0.028) and ΔPSQI (*r* = –0.38, *p* = 0.032). To demonstrate the practice effect across the 3 conditions, Figure 4 illustrates scatter plots between FC and ReHo of the specific region lMFG in the MBSR group, which highlights the relationships between distant and local connectivity during the mindfulness practices. Before the MBSR training (Figure 4A), FC and ReHo only presented non-significant correlations across the 3 conditions (*r* < 0.3, *p* > 0.05); however, after the MBSR training (Figure 4B), the FC_*dACC–lMFG*_ presented positive correlation with ReHo in both *Breathing* (*r* = 0.68, *p* = 0.002) and *BodyScan* (*r* = 0.51, *p* = 0.029), but not in the resting state (*r* = –0.15, *p* = 0.56).

## 4. Discussion

Most studies employing mindfulness-based interventions have focused on overall changes in participants’ subjective perceptions and objective neurophysiology or immunology (Black and Slavich, 2017), and few have identified the brain functional differences across different mindfulness practices (Lumma et al., 2015; Kok and Singer, 2017); However, each mindfulness practice yields differential psychological characteristics and changes in brain function. Therefore, this study investigated the differences among resting, breathing and body scan states in terms of brain connectivity. The results of both the questionnaires and brain FC indices suggested that the practices of *breathing* and *body scan* over the 8-week MBSR program allowed the participants to hone their interoceptive awareness. In the questionnaire results, the increased FFMQ scores indicated enhanced mindfulness level, and the reduction in DERS and PSQI scores indicated improvements in emotional regulation and sleep quality. The FC indices (both FC and ReHo) effectively reflected the effects of the mindfulness training as well. Specifically, the SN-FC linked to different brain regions between breathing and body scan, which was particularly pronounced after the MBSR program (Figure 1). In the traditional mindfulness training, the mindful breathing involves the single-point fixated attention to the exhalation/inhalation, either on the nostrils or abdomen, without shifting the target. Body scan, in contrast, involves the continuous attention shift on the physical sensation, from one body part and to the next. Both of them require the attention on the body sensations, but the distinction between breathing and body scan might be from the attention shift in the body scan, which might be the reason that the more engagement between SN and the lMFG (part of DLPFC) in the body scan, instead of the breathing. We also compared the association between FC and ReHo and identified the underlying information processing patterns of the resting state and the two mindfulness practices. After the MBSR program, the elevated FC and the sustained ReHo were observed during both breathing and body scan compared to the resting state, indicating increased distant connectivity along with maintenance of local connectivity. This result supports the conjecture that a focus on mindfulness task coincides with an increase in long-distance communications (especially on between-network connectivity) without sacrificing the local communications (Figures 2, 3). After the MBSR training, the stronger correlation between FC and ReHo at the body scan compared with at the resting state (Figures 3, 4) indicates that the body scan exercises the SN into an alternative information processing pattern. To assess the time/training effect, we found that the altered FC or ReHo were significantly associated with the score changes of Observe, implying that the elevated connectivity, in certain brain regions, contributed to the interoceptive perception and emotion regulation. Although both mindfulness practices are based on interoceptive awareness, the emphasis enhances both long-distance and local information connectivity in the on specific bodily sensations (breathing and body scan) yielded distinct spatial patterns of the SN connectivity, contributing to different facets of mindfulness. As compared with the normal resting state, both interoceptive mindfulness practices changed pattern of brain communication (distant-local in Figure 4), but this difference was only apparent after MBSR program. The functional specificity of mindfulness practices offers a novel viewpoint on the altered information processing across mindfulness practices.

### 4.1. MBSR training effect

In standard MBSR programs, practitioners are taught the breathing and body scan practices at the beginning of the formal practice phase, indicating that these are fundamental techniques for honing attention and interoceptive awareness. The body scan practice involves attentional shifts to multiple body parts, whereas the breathing practice encourages participants to focus on a single bodily region. At the outset, when none of the participants had prior knowledge of the two mindfulness techniques, their brain function indices suggested no functional changes associated with either technique relative to the resting condition. This was observed in both the preprogram MBSR group (Figure 4) and the CTRL group. However, after the 8-week program, significant differences in the functional effects of breathing and body scan in terms of the SN connectivity were observed. SN exhibited enhanced internetwork connectivity with the posterior brain during the breathing practice and increased connectivity with the frontal lobe during the body scan practice (Figure 1). By contrast, our results did not reveal any change in SN connectivity during the resting state, and it remained the same in the POST session (Figure 2). These observations demonstrate the link between enhanced functional connectivity and increased interoceptive awareness.

This specificity was only observable after long-term practice. During the breathing practice, dACC showed enhanced internetwork connectivity with the occipital regions (ROI 2a). These results may be attributable to the involvement of visual imagery perception when novice participants tend to envision airflow through their nostrils (Ganis et al., 2004; Haruna et al., 2019; Weaver et al., 2020). By contrast, during the body scan, enhanced internetwork FC between dACC and lMFG (ROI 2b) and between insula and the SMN (ROI 2c) was observed. The enhanced dACC-lMFG connectivity might reflect the attention engagements toward the interoceptive awareness, and the insula–SMN connectivity might be attributable to the interaction between interoceptive awareness and bodily sensations (Blatow et al., 2007; Ferri et al., 2012). Although we did not conduct counterbalancing in our experiment of mindfulness practices, the spatially distinctive changes in connectivity substantiated our presumption that the two mindfulness techniques yield different effects at the neurophysiological level. Subsequent studies should identify subtle differences between these two mindfulness techniques in a counterbalanced order.

### 4.2. Information processing between breathing and body scan in the MBSR group

In this study, the two brain functional metrics, FC and ReHo, were adopted to investigate underlying information processing mechanisms from the perspective of distant and local neural communications, respectively. After the MBSR program, although an elevated FC was observed in SN, ReHo increased only in the inferior parietal lobe during the body scan practice. Hence, long-distance connectivity increased during the mindfulness practices, while local connectivity remained the same. The results indicate that the pattern of information processing in the brain shifted toward one of the enhanced internetwork communications with the preservation of local information communication during both practices. Studies implementing meditation training programs of similar durations reported decreased ReHo during the postintervention testing (Xiao et al., 2019; Zhao et al., 2019). Our results suggest that the ReHo values remained largely unchanged during the mindfulness practices. However, Figure 4 highlights strong association between FC and ReHo (FC-ReHo correlation) in the lMFG after training. Compared with the resting state, the stronger positive FC-ReHo correlations during the breathing and body scan practices indicates simultaneous increases in distant and local connectivity during the mindfulness practices, with levels varying by participant. Steiger’s Z test revealed stronger FC-ReHo correlation in the dACC-lMFG connectivity during the mindfulness practices compared with those recorded during resting (*p* = 0.01 and 0.03 for *BodyScan* and *Breathing*, respectively). FC-ReHo correlations were only discernible after the 8-week MBSR program, and no significant FC-ReHo correlation was observed during the mindfulness practices (breathing and body scan) before program attendance. Although the FC-ReHo correlation varied by participant, these findings provide preliminary evidence that mindfulness practices alter information processing associated with SN connectivity in various brain regions. Future studies should investigate the functional changes specific to other types of mindfulness techniques and brain networks.

Previous literature mentioned that longitudinal mindfulness training is related with the most prominent three networks—SN, default-mode network (DMN), and executive control network (ECN) (Sezer et al., 2022), but it was not reported for the practice-specific FC changes after mindfulness training. In this work, we targeted on the SN because the mindfulness practices—breathing and body-scan—are related to the interoception (the function of SN), instead of the mind-wandering (the function of DMN) and goal-oriented execution (the function of ECN). Even though, the other two networks were also evaluated in both mindfulness practices and training. The DMN-FC presented significant training effects on the FC_PCC–superior temporal_ and FC_insula–PCC_, but there was a lack of significance finding in the between-condition comparisons (see Supplementary Figure 1 in Supplementary material), but neither the training effect nor the condition effect was found in the ECN-FC. The functional roles of DMN and ECN in mindful breathing and body-scan await further investigations.

### 4.3. Limitations

This study had several limitations. First, the sample size was small and may have had insufficient statistical power because of the two-group interventional design. Relatively, the CTRL group exhibited no significant longitudinal differences in both behavioral and brain functional indices over the 8-week study period, hence we did not present their imaging findings, so as the between-group comparisons in the imaging data. The between-group comparisons of the questionnaires were shown in Supplementary Table 1, in which we found that the MBSR group showed higher mindfulness scale and marginally lower difficulty in emotion regulation compared to the CTRL group after MBSR training. Studies with more sample size are warranted to verify the practice specificity. Second, although the total home-based practice times of all MBSR group were recorded (mean ± SEM: 43.3 ± 0.7 h), we did not identify a relationship between practice time and the changes in FFMQ score (*r* = 0.18, *p* = 0.47) or changes in the SN connectivity (|*r*| < 0.39, *p* > 0.11). This might be attributable to the small sample size or considerable between-individual differences. Third, we conducted the *Resting*, *Breath* and *BodyScan* conditions in a fixed order because most of the mindfulness practitioners would start with the breathing and then proceed to the body scan practice. In this study, the practitioners were able to sufficiently engage in the appropriate mindfulness practice. However, without a counterbalanced design, separating the effects of the breathing practice from those of the body scan practice was difficult. Nevertheless, our findings indicate spatial differences in the connectivity patterns of these practices (Figure 1) after the MBSR training, which supports our hypothesis that each mindfulness practice affects brain functions distinctively. Subsequent studies can improve the experimental design by including a brief period of breathing at the start of all mindfulness practices to address the practice interaction problem.

## Data availability statement

The raw data supporting the conclusions of this article will be made available by the authors, without undue reservation.

## Ethics statement

The studies involving human participants were reviewed and approved by Taipei Medical University Joint Institute Review Board (TMU-JIRB, N201905049). The participants provided their written informed consents to participate in this study.

## Author contributions

CW and C-MH: conception and experiment design. F-YH, Y-TC, and H-YN: mindfulness training and data collection. S-FG and Y-PC: data analysis. S-FG, Y-PC, C-FH, and C-HC: preparation of the manuscript. All authors reviewed and approved the manuscript.
