# Supplementary material for: Interoceptive awareness: MBSR training alters information processing of salience network
Source: Front Behav Neurosci. 2023 Mar 21;17:1008086. doi: 10.3389/fnbeh.2023.1008086 (PMC10070746; doi:10.3389/fnbeh.2023.1008086)
Supplement: Supplementary file 1 [file Data_Sheet_1.PDF]

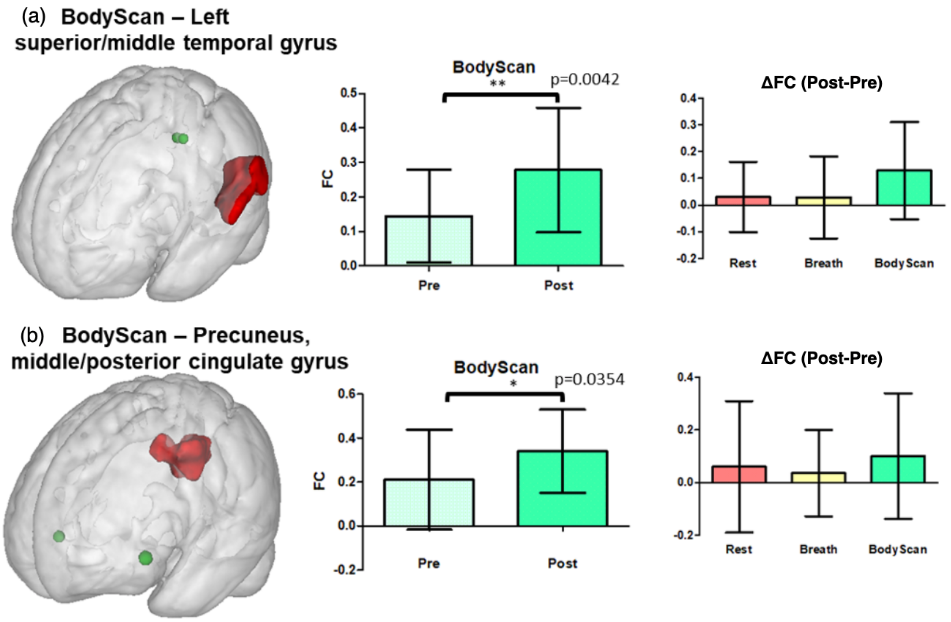

Figure S1 After MBSR training, (a) FC between PCC and left superior/middle temporal gyrus increased significantly during the body-scan practice ($p < .05$); (b) FC between bilateral insula and middle/posterior cingulate cortex showed significant increase during the body-scan practice ($p < .05$). These two FC findings were related with the default-mode network; however, rm-ANOVA of ΔFC (Rest, Breath and BodyScan) did not revealed significant differences between conditions ($p < .05$).

Table S1. Questionnaire scores of the between-group comparisons (MBSR vs. CTRL)

Pre-test

	MBSR	CTRL	Statistical analysis
FFMQ (total)	114.9 (16.99)	116.6 (17.05)	p = 0.889 (t_{30} = -0.140)
DERS	97.78 (20.72)	95.5 (23.24)	p = 0.711 (t_{30} = 0.374)
PSQI	8.389 (3.852)	5.571 (2.901)	p = 0.034* (t_{30} = 2.226)

Post-test

	MBSR	CTRL	Statistical analysis
FFMQ (total)	142.6 (23.24)	115.9 (15.2)	p < 0.001*** (t_{30} = 4.220)
DERS	84.72 (23.21)	99.93 (16.10)	p = 0.062 (t_{30} = -1.941)
PSQI	6.111 (3.848)	5.000 (2.801)	p = 0.403 (t_{30} = 0.849)

Note: Mean (standard deviation); *p<0.05; **p<0.01; ***p<0.001; n.s.: non-significant
